# Outer membrane protein genes and their small non-coding RNA regulator genes in *Photorhabdus luminescens*

**DOI:** 10.1186/1745-6150-1-12

**Published:** 2006-05-22

**Authors:** Dimitris Papamichail, Nicholas Delihas

**Affiliations:** 1 Department of Computer Sciences, SUNY, Stony Brook, NY 11794-4400, USA; 2 Department of Molecular Genetics and Microbiology, School of Medicine, SUNY, Stony Brook, NY 11794-5222, USA

## Abstract

**Introduction:**

Three major outer membrane protein genes of *Escherichia coli*, *ompF*, *ompC*, and *ompA *respond to stress factors. Transcripts from these genes are regulated by the small non-coding RNAs *micF*, *micC*, and *micA*, respectively. Here we examine *Photorhabdus luminescens*, an organism that has a different habitat from *E. coli *for outer membrane protein genes and their regulatory RNA genes.

**Results:**

By bioinformatics analysis of conserved genetic loci, mRNA 5'UTR sequences, RNA secondary structure motifs, upstream promoter regions and protein sequence homologies, an *ompF *-like porin gene in *P. luminescens *as well as a duplication of this gene have been predicted. Gene loci for *micF *RNA, as well as OmpC protein and its associated regulatory *micC *RNA, were not found. Significantly, a sequence bearing the appropriate signatures of the *E. coli micA *RNA was located. The *ompA *homolog was previously annotated in *P*. *luminescens*.

**Conclusion:**

Presence of an *ompF*-like porin in *P. luminescens *is in keeping with the necessity to allow for passage of small molecules into the cell. The apparent lack of *ompC*, *micC *and *micF *suggests that these genes are not essential to *P. luminescens *and *ompC *and *micF *in particular may have been lost when the organism entered its defined life cycle and partially protected habitat. Control of porin gene expression by RNA may be more prevalent in free- living cells where survival is dependent on the ability to make rapid adjustments in response to environmental stress. Regulation of *ompA *by *micA *may have been retained due to a necessity for *ompA *control during one or both stages of the *P. luminescens *life cycle.

**Reviewers:**

This article was reviewed by Tal Dagan (nominated by Dan Graur), Mikhail Gelfand and Anna Gerasimova (nominated by Mikhail Gelfand) and J Peter Gogarten.

**Open peer review:**

Reviewed by Tal Dagan (nominated by Dan Graur), Mikhail Gelfand and Anna Gerasimova (nominated by Mikhail Gelfand) and J Peter Gogarten. For the full reviews, please go to the Reviewers' comments section.

## Background

Outer membrane porin proteins allow for the passive diffusion of small solutes into the bacterial cell. Passage of molecules through the cell envelope and control of this process are crucial to cell survival when nutrients are scarce or when the cell is exposure to toxins or other adverse conditions. In *E. coli *and related gamma-proteobacteria, the major outer membrane porin proteins are OmpF and OmpC [1]. *ompF *and *ompC *genes are regulated transcriptionally by transcription factor OmpR in response to osmolarity change in the environment [2]. *ompF *is also regulated post-transcriptionally at the level of messenger RNA stability by the *trans*-encoded antisense RNA *micF *in response to various environmental factors such as temperature increase, oxidative stress and exposure to toxic compounds [3]. Regulatory non-coding RNAs (ncRNAs) in prokaryotes are also referred to as *trans*-encoded antisense RNAs. *ompC *in *E. coli *is regulated post-transcriptionally by the regulatory ncRNA *micC *[4]. OmpA, another major outer membrane protein, has multiple and more complex functions [5] For example, OmpA adds to the stability of the cell envelope by linking the outer membrane to the peptidoglycan. It is involved in bacterial conjugation [6] and functions as a porin protein as well [7]. The stability of *ompA *mRNA varies with bacterial growth rate [8] and *ompA *mRNA is degraded at a fast rate when cells enter stationary phase [9]. Udekwu et al [10] recently showed that the regulatory *micA *RNA post-transcriptionally regulates *ompA *mRNA. In addition, *micA *is induced at stationary phase, a stress condition [10]. Thus in *E. coli*, three major outer membrane proteins, OmpF, OmpC, and OmpA are all regulated by specific small RNAs in response to stress factors.

*Photorhabdus luminescens *is phylogenetically a member of the gamma- proteobacteria based on analyses of 50 gamma proteobacterial 16S rRNA genes [11]. In a phylogenetic tree based on the *sctV *gene (which encodes a highly conserved inner membrane protein), *P. luminescens *falls into the *Yersinia *family [12].

*P. luminescens *has a complex life cycle and proliferates in two distinctly different environments [13,14]. *P. luminescens *lives symbiotically in the nematode gut, but also has a pathogenic phase when the worm, which normally resides in the soil, infects an insect. In this stage, *P. luminescens *cells are released into the circulatory system (hemocoel) of the insect by the nematode. Here the bacteria grow and commence with the rapid killing of the insect and both the nematode and the bacteria feed from the dead insect [13,14]. After nutrients derived from the insect carcass are depleted, the bacteria re-associate with the nematode and the symbiotic relationship is re-established. *P. luminescens *has not been found as a free living organism and thus differs significantly from *E. coli *and most other closely related gamma-proteobacteria.

During the evolutionary period when *P. luminescens *evolved into a symbiont and a pathogen, its genome expanded such that it has one of the largest chromosomes of the gamma-proteobacteria (~5.7 Mb) [15]. This expansion is related to its pathogenic phase [13-15]. However certain genetic elements that contribute towards survival in a harsh environment but are no longer needed may have been lost from the genome during evolution of the organism.

Using a bioinformatics approach, the *P. luminescens *genome was analyzed for outer membrane porin protein and associated regulatory RNA genes. We find a limited presence of the porin genes and their RNA regulators.

## Results

### Analyses of *Photorhabdus omp*-like genes

#### Gene arrangements: *ompF *locus

**Figure 1:**
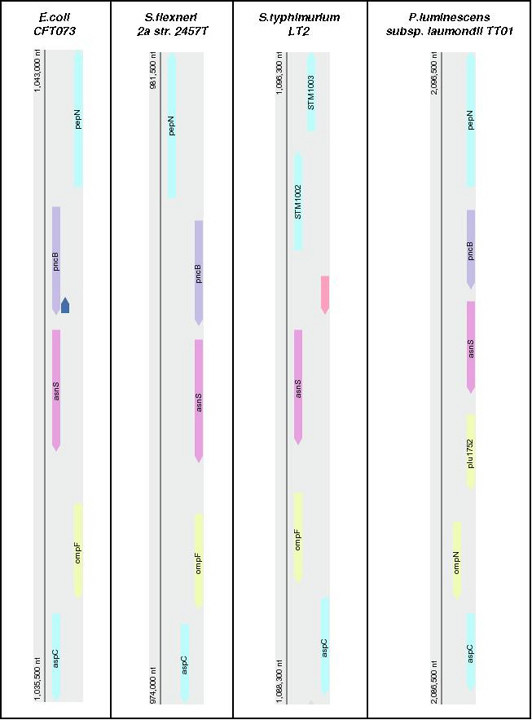
*ompF *gene locus in *Escherichia coli CFT073, Shigella flexneri 2a str. 2457T, Salmonella typhimurium LT2*, and in the *Photorhabdus luminescens subsp. laumondii TTO1 *locus with annotation as *ompN*. In the *S. typhimurium *locus, genes *pncB *and *pepN *(not shown) are present however there are three genes inserted between *pncB *and *asnS*. The unmarked blue gene in *E. coli *is a hypothetical protein 61 amino acids long. The unmarked pink gene in *S. typhimurium *is a partially characterized protein and assumed to be a putative leucine response regulator. Gene arrangements are as shown on the NCBI website [16].

Analysis of nearest neighbor genes in the gamma subdivision of proteobacteria reveals a conserved gene arrangement surrounding the porin protein gene *ompF *in species closely related to *E. coli *(Figure 1). *OmpF *is flanked on one side by *aspC*, the aspartate aminotransferase gene and by *asnS*, the asparaginyl-tRNA synthetase gene on the other side. In addition, *pncB *(nicotinate phosphoribosyltransferase) and *pepN *(aminopeptidase N) are situated to the right of the *ompF *gene locus in the schematic shown. Similar gene arrangements for the *ompF *locus are also in more distantly related organisms, e.g., *Buchnera aphidicola str. Sg*. The loci in *Yersinia *species (not shown) have the same gene arrangements, but *ompF *is referred to as a general porin gene, e.g., see *yptb1435*, *Yersinia pseudotuberculosis IP 32953, complete genome *annotation, [17]. However an *ompF *ortholog has been located in this locus in *Yersinia *species [18]. *Xenorhabdus nematophilia*, which has an *ompF *-like porin gene (*opmP*), displays the similar nearest neighbor gene arrangement as that in *E. coli *[19]. *X. nematophilia *has a similar life cycle as that of *P. luminescens*.

In *P. luminescens*, an identical nearest neighbor arrangement is present around the gene annotated as *ompN *(Figure 1). *ompN *is flanked by *aspC *and *asnS *with *pncB *and *pepN *situated adjacent to *asnS *as is found in *E. coli *and the other species shown. However, *Photorhabdus *also has a hypothetical protein gene (*plu1752*) situated between *ompN *and *asnS *(Figure 1). Thus the gene annotated as *ompN *in *P. luminescens *has the enterobacterial *ompF *chromosomal locus signature with the exception of an inserted protein gene. Analysis of nearest neighbor genes and their conservation in related enterobacterial species has been reported before, e.g., for non- coding RNA genes [20]. In addition Notebaart et al [21] have presented a detail analysis of nearest neighbor genes, protein amino acid sequence and gene function predictions.

### Characteristics of *P. luminescens ompN*

#### a. Amino acid sequence identities

A comparison of amino acid sequence identities of the *P. luminescens *OmpN with representative gamma-proteobacteria outer membrane proteins is shown in Table 1. The highest identity is with OpmP, the OmpF-like porin in *X. nematophilia*. However identities with other porin proteins in other bacteria are also high and a clear segregation of OmpF protein sequences is not apparent (Table 1). Due to evolutionary relatedness between many outer membrane proteins, there is high sequence identity between these proteins, but they are not necessarily orthologs of each other and identities between porin protein amino acid sequences is of limited use in gene annotation [18].

**Table 1:** *P. luminescens *OmpNamino acid sequence identities

**Organism**	**Porin**	Percent identity*
*X. nematophilus*	OpmP (OmpF-like porin)	68%
*Y. pseudotuberculosis*	*yptb1435 *(OmpF homolog)	62%
*S. marcescens*	OmpF	62%
*S. sonnei*	OmpC	59%
*E. coli*	OmpC	58%
*E. coli*	OmpN	57%
*S. enterica*	OmpC	56%
*S. sonnei*	OmpF	54%
*E. coli*	OmpF	54%

* Sequence identities of *P. luminescens *OmpN with other Omps from the NCBI Protein-protein BLAST (blastp) program

*ompN *is not uniformly found in the gamma-proteobacteria. For example, there is no annotation for the *ompN *gene in *Yersinia pestis *and a protein blast search using the *E. coli *OmpN protein sequence does not yield an orthologous protein in the *Y. pestis *genomic sequence. Additional characteristics of *ompN *in other species are discussed below.

#### b. mRNA 5' UTR sequences and secondary structures

mRNA 5' UTR sequences can be important markers in identifying protein coding genes [18]. In the study here, a comparative sequence analysis of 5' UTRs and a comparison of mRNA 5' UTR secondary structures were performed. All parameters investigated suggest that *ompN *is the *P. luminescens *homolog of *ompF*.

**Figure 2:**
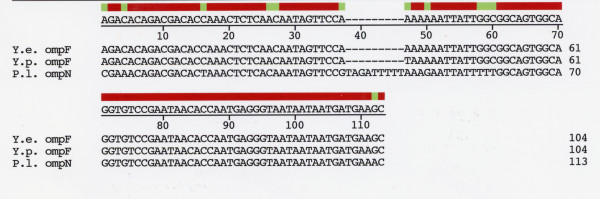
Alignment of putative *Photorhabdus luminescens ompN *mRNA 5' UTR nucleotide sequence with *ompF *mRNA 5' UTRs of *Yersinia pestis *and *Yersinia enterocolitica*. The 5' end of the *ompN *5' UTR was assigned by alignment with Yersinia and other enterobacterial *ompF *5' UTR sequences. Y.e., Y.p., and P.l. refer to *Y. enterocolitica, Y. pestis*, and *P. luminescens *sequences, respectively. The *Y. enterocolitica *sequence can be accessed in the *Y. enterocolitica *Blast Server [22]. Sequences were aligned with the DNASTAR, Inc program using ClustalW alignment.

The *P. luminescens ompN *mRNA 5' UTR was deduced from alignment of sequences upstream of the ATG coding start site with 5'UTR sequences of *Yersinia *species (Figure 2). A comparison with *Yersinia *sequences is pertinent since *Yersinia *and *Photorhabdus *species are closely related evolutionarily [11,12]. The *P. luminescens ompN *mRNA 5' UTR has a very high nucleotide sequence identity to the *ompF *mRNA 5'UTR of *Yersinia pestis *(89.5%). Interestingly, there is 100% sequence conservation at the 3' half of the 5'UTR from positions 61 – 111 (Figure 2). The *Photorhabdus ompN *5' UTR also displays a high sequence identity with other gamma-proteobacteria *ompF *5' UTRs (data not shown).

**Figure 3:**
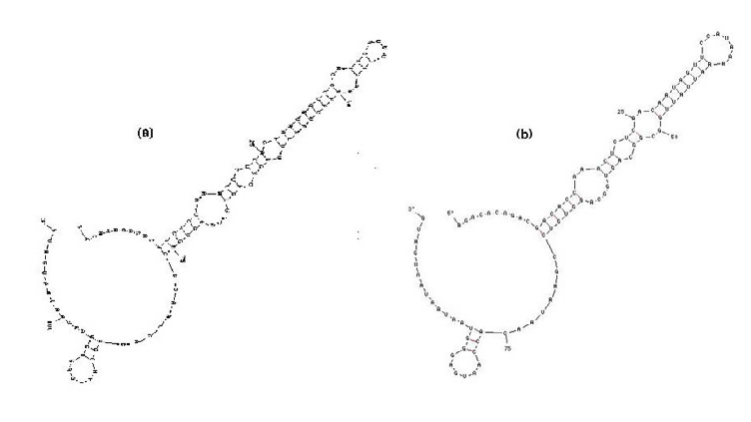
5' UTR secondary structures of a) *P. luminescens ompN *mRNA 5' UTR and b) *Y. pestis ompF *mRNA. Structures modeled by the mfold program (26).

The putative *P. luminescens ompN *mRNA 5'UTR also shares signatures of the generalized *ompF *5' UTR secondary structure. These signatures are highly specific to *ompF *mRNAs and consist of a long stem loop which starts at ~5–10 nt from the 5' end of the 5' UTR [23-25] (Figure 3). The stem contains several bulged and looped out positions. In addition, there is also a small stem loop close to the 3' end that encompasses the Shine- Dalgarno (S-D) ribosome binding site (e.g., GAGG in *E. coli *and Y *. pestis*). The *Photorhabdus ompN *mRNA 5' UTR closely conforms to the gamma-proteobacteria *ompF *mRNA 5' UTR secondary structure motif. Figure 3 shows a comparison with the *Y. pestis ompF *mRNA 5' UTR secondary structure. There are minor differences in the upper portion of the large stem loop, however base pairing differences are characteristic of *ompF *mRNA 5' UTR structures [24]. Secondary structures of *E. coli ompC*, *ompN *and other outer membrane protein mRNA 5' UTRs do not display the *ompF *5' UTR signatures.

#### c. Upstream regulatory sequences

**Figure 4:**
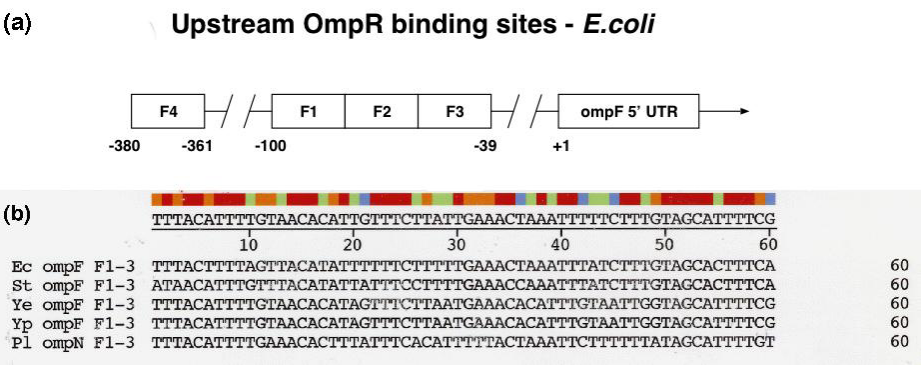
a) Schematic of OmpR binding sites F1–4 located upstream of *ompF *in *E. coli *(29); b) Alignment of OmpR binding sites F1, F2, and F3 upstream of *ompF *in enterobacteria including the putative site upstream of *Photorhabdus ompN*. Ec, St, Ye, Yp, and Pl refer to *E. coli *S. typhimurium, *Y. enterocolitica, Y. pestis*, and *P. luminescens *sequence, respectively. The putative F 1–3 sites in *P. luminescens *were deduced by the alignment of sequences upstream of the ATG translational start site of *ompN *and of *ompF *from the four enteric bacteria shown. Binding sites are as follows: F1, positions 1–20; F2, positions 21–40; F3, positions 41–60. Note: two nt positions between F2 and F3, which are not part of F1–3 binding sites (29) are not included in the figure.

Another important signature of *ompF *is the conservation and specificity of its upstream regulatory sequences. OmpR, a transcription factor found in *E. coli *and related organisms, is part of the two-component signal transduction regulatory locus *ompB *[27,2,28]. OmpR transcriptionally regulates expression of *ompF *and *ompC *genes in response to osmolarity change in *E. coli *and related species. Four OmpR binding sites F1–4 are located upstream of *ompF *in *E. coli *[29,30] (Figure 4a). Figure 4b shows an alignment of OmpR binding sites F1, F2, and F3 upstream of *ompF *from several species, including the proposed site upstream of *P. luminescens ompN*. F 1–3 sites in *P. luminescens *were deduced by the alignment of sequences upstream of the ATG translational start site of *ompN *and of *ompF *from the four enteric bacteria shown. The *E. coli *sites as described by Bergstrom et al [29] were used as a base line. These sites are involved in transcriptional activation in *E. coli*. There is a comparable sequence identity between *Photorhabdus *and the three enterobacteria *ompF *upstream sites F1–3 (Table 2). In addition, most nucleotide positions crucial for OmpR binding in *E. coli *[31,32] are conserved in *P. luminescens *sites, e.g., A_4 _C_5 _and A_14_C_15 _in F1, A_34_, C_35 _in F2, and C_53 _in F3. Conservation of these crucial positions suggests that the *P. luminescens *sites function in OmpR binding. A GNNNC motif found in OmpR binding elements [32] is present in the *P. luminescens *sequence, G_11_AAAC_15_, albeit there is only one copy which is in the F1 site (Figure 4b).

**Table 2:** Percent Identity F1–3 binding sites

	***E.coli***	***S.typhimurium***	***Y.pestis***	***Y.enterocolitica***	***P.luminescens***
*E.coli*		86.7	70.0	70.0	66.7
*S.typhimurium*			65.0	65.0	63.3
*Y.pestis*				100.0	66.7
*Y.enterocolitica*					66.7

**Figure 5:**
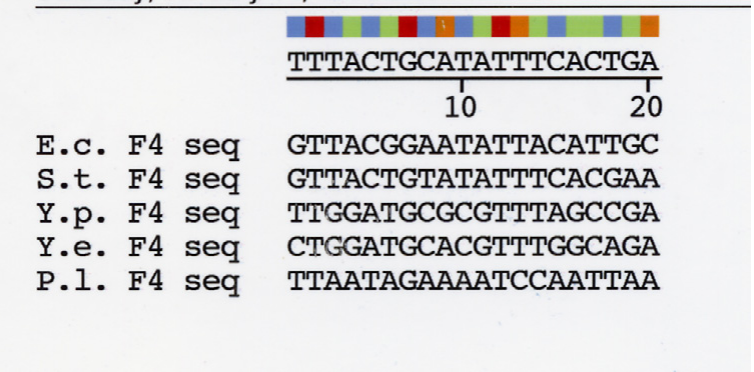
Nucleotide sequence upstream of *ompN *in *P. luminescens *aligned with F4 binding site sequences in four enterobacterial species. Sequences identified by alignment of upstream sequences as described in Figure 4 caption.

The F4 site differs from F1–3 in that it is involved in repression and not activation of *ompF *expression [29]. An hypothetical F4 site was also located upstream of *ompN *in *Photorhabdus *based on alignment of sequences with the nt positions and sequence described for the *E. coli *F4 [29] (Figure 5). However, F4 sequences have diverged and are more complex than sites F1–3, e.g., *Y. pestis *and *Y. enterocolitica *species reveal low identity with the other gamma-proteobacteria (e.g., 28.6% and 33.3% identity respectively, compared with the *E. coli *F4 site) (Table 3). Although this is close to random identity, the *Yersinia *species have a GNNNC motif. The identity between *Photorhabdus *and the *E. coli *F4 sites is much higher, 52.4%, however the putative *P. luminescens *F4 lacks the GNNNC motif (Figure 5). Thus, it is unlikely that this sequence functions as a repressor of the proposed *P. luminescens ompF*.

**Table 3:** Percent Identity F4 binding site

	***E.coli***	***S.typhimurium***	***Y.pestis***	***Y.enterocolitica***	***P.luminescens***
*E.coli*	***	**66.7**	**28.6**	**33.3**	**52.4**

#### d. OmpF loop 3

Structurally, OmpF consists of trans-membrane beta strands and eight loops. Loop 3 (L-3) of OmpF is critical in formation of the channel pore and conductance and thus is a major functional determinant of the porin protein [33,34]. The amino acid sequence of L-3 of the proposed *P. luminescens *OmpF-like porin was aligned with L-3 sequences from related bacteria (Figure S1, ,Supplement, Additional file 1). The *Photorhabdus *sequence displays a high identity to the L-3 loop sequences of *X. nematophilia *OpmP (88% identity) and the *Yersinia *OmpF L-3 (82%) (Table S1, Supplement, Additional file 1). The sequence identity to the *E. coli *OmpN L-3 is 68%. Interestingly, there are substantial differences between the other gamma- proteobacterial OmpF L-3, especially between *E. coli *and *Salmonella *(49% Identity) (Table S1, Supplement, Additional file 1). This suggests a complex selection pressure in amino acid sequence in related gamma enterobacteria and possible structure-function differences of this critical loop. An L-3 phylogenetic tree is in Figure S2, Supplement, Additional file 1).

In summary, a) high identity with *ompF *mRNA 5' UTR sequences, b) shared mRNA 5' UTR secondary structure motifs, c) similarities between neighbor genes in the *P. luminescens ompN *locus and genes of the gamma-proteobacteria *ompF *locus, d) high sequence identities in upstream transcriptional regulatory sites F1–3, and e) identity of OmpF loop L-3 amino acid sequences all suggest that the annotated *ompN *gene is an *ompF*-ortholog in *P. luminescens*.

### plu1752

**Figure 6:**
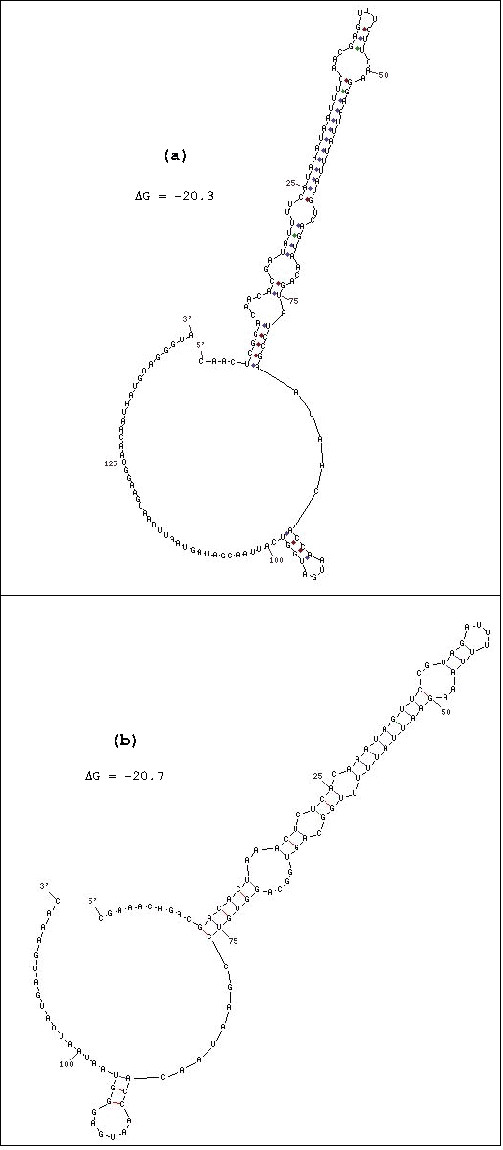
a) Deduced *plu1752 *mRNA 5'UTR secondary structure by mfold. Structure shown is alternate structure 4 from mfold (mfold structure 1 displays minor base pairing within the 28 bp insert but no differences in stem-loop structure), b) *P. luminescens ompF *mRNA 5' UTR secondary structure model.

*plu1752 *is situated between *ompN *and *asnS *in the annotated *P. luminescens *genome (Figure 1). The coding nucleotide sequence of *plu1752 *shows no frameshift mutations, no insertions, and one triplet deletion when sequences are compared with the proposed *P. luminescens ompF *(data not shown). Thus *plu1752 *encodes a nearly full length protein compared with OmpF. *plu1752 *has a 59% amino acid sequence identity to the *P. luminescens *OmpF-like porin and 59% identity to the *X. nematophilia *OpmP. Its identity with *E. coli *and *Yersinia pestis *OmpC proteins is 55% and 51% respectively, and 51% to OmpS1, a porin protein gene (distinct from *ompF *and *ompC*) in *Salmonella typhi str. CT18*. The amino acid sequence identity of *plu1752 *is closest to OmpF-related proteins but only slightly higher than to OmpC proteins. On the other hand, the *plu1752 *deduced mRNA 5'UTR sequence displays a high identity with that of the proposed *ompF *5' UTR *ompF *5' UTR (76.4%). Interestingly, the *plu1752 *5'UTR sequence also has a 28 bp insertion in the 3' region. However, the proposed *plu1752 *mRNA 5'UTR secondary structure closely resembles that of the *P. luminescens ompF *5' UTR (Figure 6) and the 28 base insertion between positions 92–93 of the *ompF *5' UTR does not appear to perturb the large stem loop, the primary *ompF *5' UTR structural motif. The *plu1752 *5'UTR maintains the four looped out/bulged positions present in *ompF *5' UTR major stem loop, albeit there are nucleotide sequence changes in these regions. Importantly, there are several base pair compensatory changes in stem sequences, e.g., in the stem closest to the top loop there is a change of the pairing U_38_-A_47 _(*ompF*, Figure 6b) to G_40_-U_48 _(*plu1752*, Figure 6a). This is strong evidence for evolutionary relatedness. The *plu1752 *mRNA 5'UTR displays poor sequence identity to the enteric *ompC *mRNA 5' UTRs (e.g., 47% and 36% to *Y. pestis *and *E. coli*, respectively) thus making it unlikely that it is an otholog of *ompC*. However, there is an apparent absence of an *ompC *gene in *P. luminescens *(see below).

**Figure 7:**
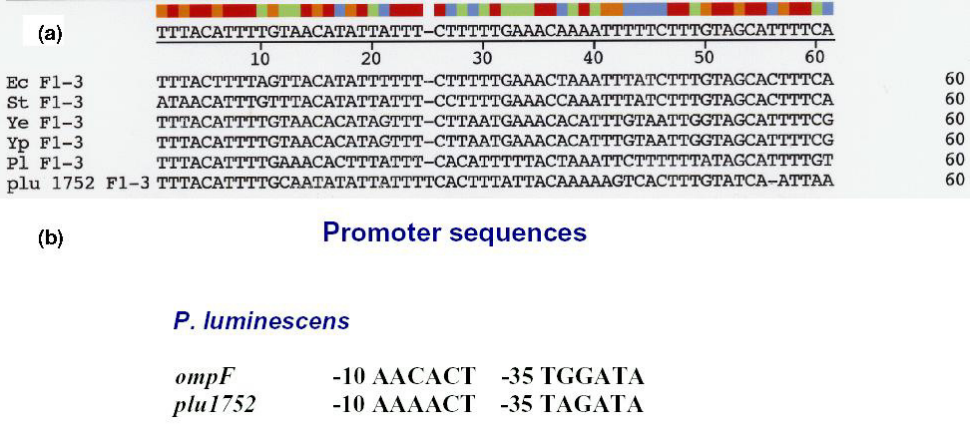
a) Alignment of upstream sequences of *plu1752 *with the F1–3 regions of enterobacterial *ompF*. Boundaries of F1, 2, 3 as in Figure 4b caption; b) comparison of -10 and -35 promoter sequences of *plu1752 *and *P. luminescens ompF*.

Comparison of the *plu1752 *upstream region with regions upstream of enterobacterial *ompF *genes reveals a close similarity with OmpR binding sites F1–3 (Figure 7a). Deduced F1–3 sequences of *plu1752 *show 70% identity with F1–3 sites of the proposed *P. luminescens ompF*. In addition, several base positions highly critical for OmpR binding (as described for Figure 4b) are also conserved in the *plu1752 *sites (Figure 7a) but also noted is the presence of only one GNNNC motif (G_50_TATC_55_, in F3). The upstream regulatory region of *plu1752 *is, however truncated, i.e., there are only 142 bp between *plu1752 *and its upstream gene, *asnS *gene. Thus, *plu1752 *appears to have no potential for an F4 binding site. The *plu1752 *-10 and -35 promoter sequences are nearly identical to those of proposed *P. luminescens ompF *(Figure 7b).

Loop 3 of Plu1752 protein has a high amino sequence identity with the proposed *P. luminescens *OmpF-like porin (77%) (Table S1, Supplement, Additional file 1). The identity with representative gamma proteobacterial OmpF L-3 sequences is much less and ranges from 44%- 59%, with the exception of sequences of *Xenorhabdus *(68%) and *Yersinia *(71%) (Table S1, Supplement, Additional file 1).

**Figure 8:**
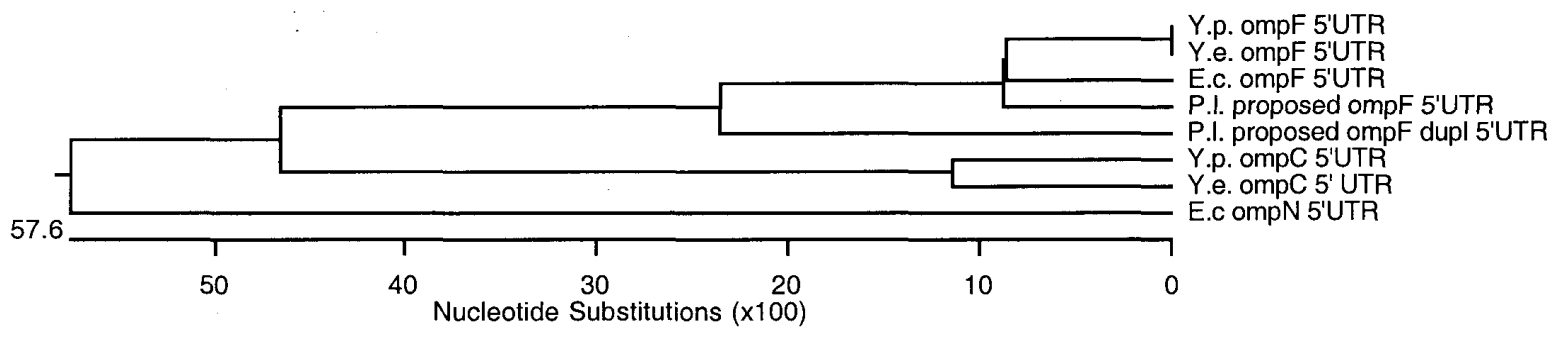
Phylogenetic tree of outer membrane protein mRNA 5' UTRs. Alignment and phylogenetic tree of outer membrane protein 5' UTRs was performed with the DNAStar ClustalW sequence alignment program.

Judging by the strong similarities in signatures between *plu1752 *and the proposed *P. luminescens ompF*, *plu1752 *may have arisen by a duplication of the *ompF*-like porin. A phylogenetic tree of the *P. luminescens *proposed *ompF *and *ompF *duplication 5' UTR sequences with representative omp 5' UTRs of other bacteria is shown in Figure 8. The *P. luminescens ompF *and *ompF *duplication sequences form a cluster with *E. coli*/*Yersinia sp. ompF *5' UTR sequences and removed from *ompN *and *ompC *5' UTRs.

A comparison of *plu1752 *characteristics with *ompF *is in Table 4. In view of the large number of non- synonymous mutations (changes in amino acids), i.e., 59% identity in amino acid composition between *P. luminescens ompF *and *plu1752*, the proposed duplication may have been an ancient one. *S. typhimurium *and *E. coli *separated about 140 million years ago and the percent amino acid changes between the *plu1752 *protein and the proposed *P. luminescens *OmpF are about the same as those between *S. typhimurium *and *E. coli *OmpF proteins (Table 4). However, further phylogenetic analyses are needed to better understand the proposed duplication event.

**Table 4:** Comparison of *ompF*-like porin and *plu1752*

***P. luminescens***	**Sequence identity**
*ompF/plu1752 *OmpF amino acid sequence	59%
*ompF/plu1752 *OmpF Loop L-3 sequence	77%
*ompF/plu1752 *nt coding sequence	68%
*ompF/plu1752 *mRNA 5' UTRs	77%
*ompF/plu1752 *F1–3 binding sites	71%

***S. typhimurium/E. coli***	
*S. typhimurium/E. coli *OmpF amino acid sequence	58%
*S. typhimurium/E. coli *OmpF Loop L-3 sequence	49%
*S. typhimurium/E. coli ompF *nt coding sequence	65%
*S. typhimurium/E. coli ompF *mRNA 5' UTRs	94%
*S. typhimurium/E. coli *F1–3 binding sites	87%

### Porin protein gene *ompC *and regulatory RNA genes *micF*, *micC *and *micA*

#### a. *ompC *and *micF*

*ompC*, which codes for the smaller porin protein was not detected in *P. luminescens*. For example, *ompC *nearest neighbor genes such as *ubiG*, *gyrA*, *rcsC*, and *rcsB *are grouped together in *E. coli *and *Yersinia pestis *and are also conserved as a group in *P. luminescens*, but the *Photorhabdus *locus does not show an annotated *ompC *[15]. Blast searches of the *P. luminescens *genome using the *ompC *coding sequence, the *ompC *mRNA 5' UTR sequence, and the *ompC *amino acid sequence from *E. coli *and *Y. pestis *did not yield sequences orthologous to *ompC *(data not shown). In addition, various other *E. coli *porin protein sequences were employed to search the *P. luminescens *genome using the NCBI protein tblastn program, however no other porin protein sequences were detected with the exception of the proposed *ompF *and *ompF *duplication *plu1752*.

In *E. coli *and related species, the *micF *gene is an independent transcriptional unit with its own promoter and it does not overlap other genes [35]. However, *micF *and *ompC *are in the same chromosomal locus and are linked by a regulatory region (253 bp in *E. coli*) that includes binding sites for the transcription factor OmpR [2]. These serve as sites for both *micF *and *ompC *transcriptional activation by OmpR [36]. This linkage region is conserved in all enterobacteria known to contain a *micF *gene [18]. The region is complex and has binding sites for eight transcription regulators of *E. coli micF *[3,18,37]; also, see [38,39]. In *P. luminescens*, both the *micF *and *ompC *genes are missing from the chromosomal locus where they normally are found. *micF *may have been transposed to another region of the chromosome, however blast searches with *micF *gene sequences from *E. coli *and *Yersinia *species, as well as with the conserved 253 nt regulatory region did not reveal homologous sequences in the *P. luminescens *genome.

**Figure 9:**
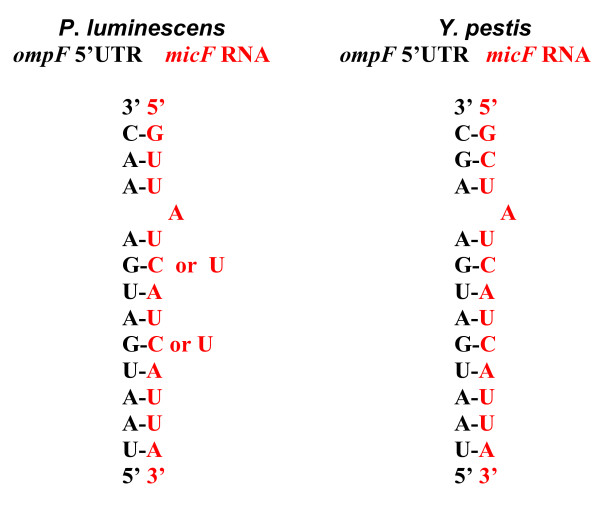
(left) Hypothetical *P. luminescens ompF *mRNA 5' UTR/*micF *RNA duplex and (right) *Y. pestis ompF *mRNA 5' UTR/*micF *RNA duplex at the 3' *ompF *UTR – 5' *micF *end interface. The C or U changes in the *P. luminescens *hypothetical *micF *RNA (left) were part of the sequences variations used in searches.

Strategies employed to further search the *P. luminescens *genome for *micF *were based on conserved portions of the *micF *sequence and phylogenetically conserved *micF *RNA/*ompF *mRNA 5'UTR duplex structural motifs. In all organisms where *micF *RNA is found (more than 6 species), there is a total conservation of the13 nt sequence at the 5' end [18]. This sequence is ^5'^GCUAUCAUCAUUA^3' ^and it forms a major part of the base pairing between *micF *RNA and *ompF *mRNA 5' UTR. A hypothetical *P. luminescens micF *RNA would consist of the same sequence based on the highly conserved portion of *ompF *mRNA 5'UTR (see Figure 2), with the exception of C_2 _to U_2 _change (Figure 9).

Intergenic regions (i.e., non protein coding regions) of the *P. luminescens *genome were scanned for sequences that provides a structure similar to the RNA/RNA duplex structure found in enterobacterial species. Permutations in the hypothetical *P. luminescens *5' end *micF *sequence shown in Figure 9 involved the use of four random substitutions in this sequence (in addition to the two C to U variables shown in Figure 9 left). Also, added structural constraints for the total hypothetical *micF *RNA structure used consisted of a rho-independent termination motif at 35–85 nt downstream from the 3' end of the 13 nt sequence. The *P. luminescens *genome was scanned and sequences analyzed. A match with forty- three positives was found for a putative *micF *RNA with the above mentioned constraints. These positives were further analyzed for ability to form an RNA/RNA duplex structure that is consistent with the conserved generalized *micF *RNA/*ompF *mRNA 5' UTR duplex structures of various enterobacterial sequences [18]. Five positives were found, however four of these have sequences in the 3' region of the hypothetical RNA chain that partly overlap protein coding genes and thus were discounted. The fifth sequence did not overlap a coding region and provided a similar interaction displayed by the *Yersinia *RNA/RNA duplex (Figure S3a, S3b, Supplement, Additional file 1). However a) this structure did not display the *ompF *3' end/*micF *5' end RNA/RNA duplex motif consisting of "blunt ends" (Figure 9, right) [18], and b) the percent identity of this fifth sequence with the *Y. pestis micF *sequence is low, 35.2%. Therefore this "fifth positive sequence" was also discounted as a *micF *candidate, albeit we cannot exclude that this may constitute an RNA transcript from promoter search analyses (Supplement, Additional file 1). No other intergenic sequences provided all the prerequisite RNA/RNA duplex signatures. A third type of search was performed with a hypothetical *P. luminescens micF *sequence that would form a perfect 14 bp complement to the *ompF *5'UTR 3' region (see Methods and Materials) and no positives were found in a search of the *P. luminescens *genome.

Thus, a) no *micF*-conserved 5' end 13 nt sequence (nt sequence in Figure 9, right), which has a rho -independent termination motif at less than 85 nt downstream of the 13 nt sequence, matched in the intergenic regions of *P. luminescens*, b) no *P. luminescens *intergenic sequences provide all the prerequisite signatures of the *micF *RNA/*ompF *mRNA 5' UTR duplex. In addition, the *P. luminescens *chromosomal locus where one would expect to find *micF *is partly rearranged and does not contain *micF *(or *ompC*).

If a putative *P. luminescens micF *RNA/*ompF *mRNA 5' UTR duplex structure diverged significantly from the phylogenetically conserved structures used for comparison, a *Photorhabdus micF *sequence would have been missed. Since the *P. luminescens ompF *mRNA 5'UTR has a secondary structure that closely conforms to the generalized 5' UTR structure and the 3' end of this UTR sequence that would interact with a hypothetical *micF *RNA is, with the exception of one nt change, completely conserved, it is less likely that a *Photorhabdus *RNA/RNA duplex structure would exist that diverges significantly from the consensus. The *E. coli *and *Y. pestis *intergenic regions of the chromosome were also scanned using the above parameters and the respective *micF *sequences were detected in these controls.

#### micC

The regulatory *micC *RNA was isolated and characterized in *E. coli *and homologous *micC *sequences were detected in close relatives such as *Klebsiella pneumonie*, but not in *Yersinia *species [4]. This RNA is 109 nt in chain length and regulates *ompC *expression post-transcriptionally in response to stress conditions [4]. Since the target *ompC *gene does not appear to be present in *P. luminescens*, it appears unlikely fhat *P. luminescens *would have *micC*. However we considered the question of whether "fossil" *micC *sequences may still be present. The *P. luminescens *genome, including the genomes of *Y. pestis *and *Y. psedutuberculosis *(which were not previously shown to have *micC*), were scanned for sequences that match *E. coli micC*. Blast searches with the NCBI Discontinuous Blast Program and utilizing the *E. coli micC *sequence as the query did not yield a match.

*micC *nearest neighbor genes were analyzed as well. In *E. coli K12*, *ydpK *(pyruvate-flavodoxin oxidoreductase gene) is situated 30 bp downstream from *micC *and *ompN *is 227 bp upstream of *micC *[4]. *ydpK *has not been annotated in *P. luminescens *and a blast search of the *P. luminescens *genome using the *Y.pestis *pyruvate-flavodoxin oxidoreductase amino acid sequence yielded only minor similarities to unrelated proteins. Thus we could not locate a locus encompassing the *micC *nearest neighbor genes in the *P. luminescens *genome.

Since *Yersinia *species have an *ompC*, theirgenomes were further searched for a *micC *homolog by analyzing sequences in a putative *micC *locus. *ydpK *is present in *Y. pestis *and *Y. psedutuberculosis *but annotated as *nifJ*. A hypothetical protein (with conserved domains similar to adenine nucleotide alpha hydrolases) is the nearest downstream neighbor (the location of *micC *and *ompN in E. coli K12*). An analysis of the sequence immediately downstream of *nifJ *was made to search for a potential sequence that may represent a *micC *homolog in *Y. pestis *and *Y. psedutuberculosis*. The 275 bp sequence (positions 2662970-2663245) downstream of the end of 3' end of *nifJ *and upstream of the start of *YPT 2254 *(hypothetical protein) contains a probable rho- independent termination motif, however it did not yield significant sequence identity when aligned with the *E. coli micC *sequence (unpublished).

#### micA

Udekwu et [10] characterized a small regulatory RNA termed *micA *that post- transcriptionally controls *E. coli *OmpA synthesis. The *micA *sequence was also identified in other enterobacteria, including *Yesinia pestis *[10] but not in more distantly related organisms.

*OmpA*, the target of *micA*, has been annotated in the *P. luminescens *genome at positions 2117794-2118900 [40]. The putative *P. luminescens ompA *mRNA 5' UTR sequence was deduced from an alignment of sequences upstream of the *P. luminescens ompA *ATG start with *E.coli *and *Y. pestis ompA *mRNA 5' UTR sequences (Figure S4, Supplement, Additional file 1). The 5' UTRs of the 3 species show a divergence in sequence, particularly that of *P. luminescens*, but the region encompassing ~30 nt at the 3' end of the mRNA 5' UTR, the site that interacts with *micA *RNA in *E. coli *[10], is highly conserved and shows an identity of ~80% between *P. luminescens *and *E.coli *and *Y. pestis ompA *mRNA 5' UTR sequences.

**Figure 10:**
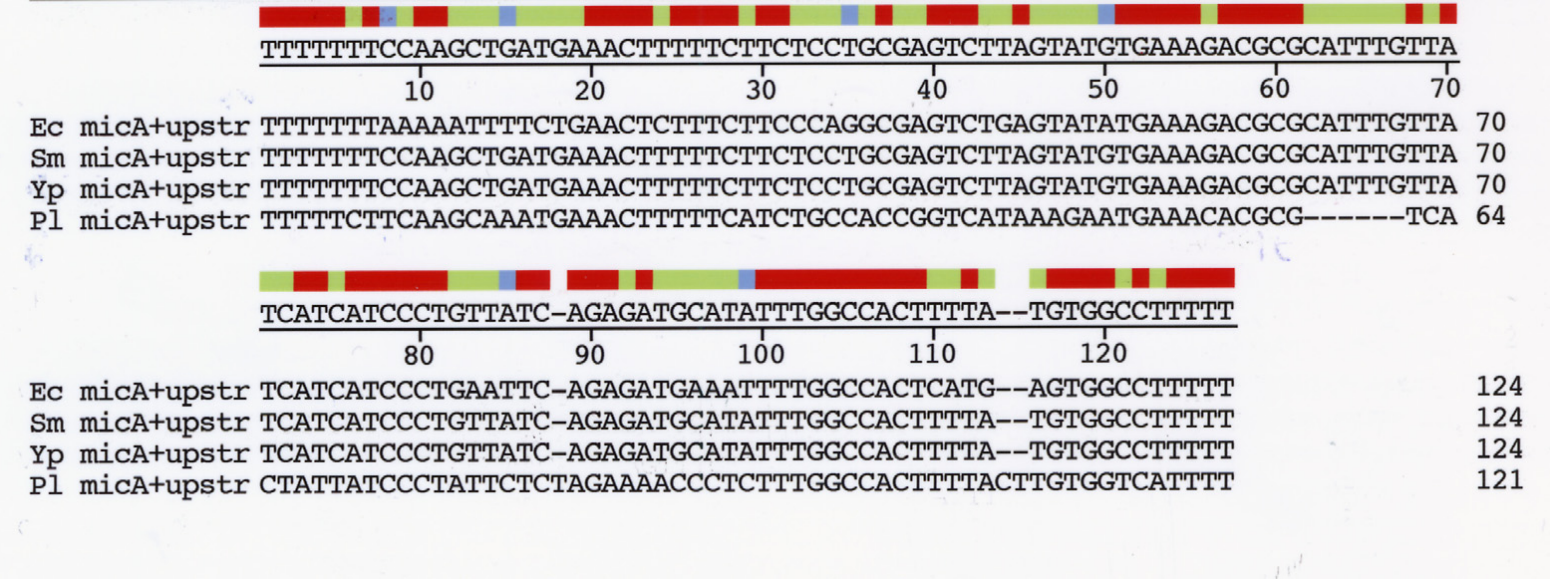
Alignment of *micA *sequences with upstream promoter region sequences. Position 52 is the 5' start of the *micA *gene and the upstream sequence shown encompasses positions 1–51. In *P. luminescens*, the putative -10 promoter site is at positions 40–45 (GTCATA sequence) and the -35 is at 16–21 (ATGAAA sequence). The *P. luminescens micA *and upstream region sequences shown in the figure are located at genomic positions 1451620-1451502.

The *P. luminescens *genome was searched for sequences similar to *micA*. A blast search using the *E. coli *and *Y. pestis micA *nucleotide sequences did not yield a match. In *E. coli *and *Y. pestis *genomes, *micA *is found between *luxS *and *gshA *[10]. To further search for the RNA gene, the nucleotide sequence between *luxS *and *gshA *in *P. luminescens *was scanned for homology to *micA *sequences. This region in *P. luminescens *was aligned with *E. coli *and *Y. pestis micA *sequences and a putative *micA *homologous sequence was identified (Figure 10). Position 52 shown in Figure 10 is the 5' start of the *micA *gene and positions 1–51 encompass the upstream promoter region. *micA *sequence identities between the four organisms shown are in the range of 58.8 – 61.8%. The 23 bp segment at the 5' end region of the putative *P. luminescens micA*, that includes sequences that would interact with the *P. luminescens ompA *5' UTR, shows a slightly higher identity of 65.2% compared with both *E. coli *and *Y. pestis micA *5' end regions. In addition, the upstream -10 and -35 promoter sequences are nearly identical between *P. luminescens *and *Y. pestis *(Figure 10).

**Figure 11:**
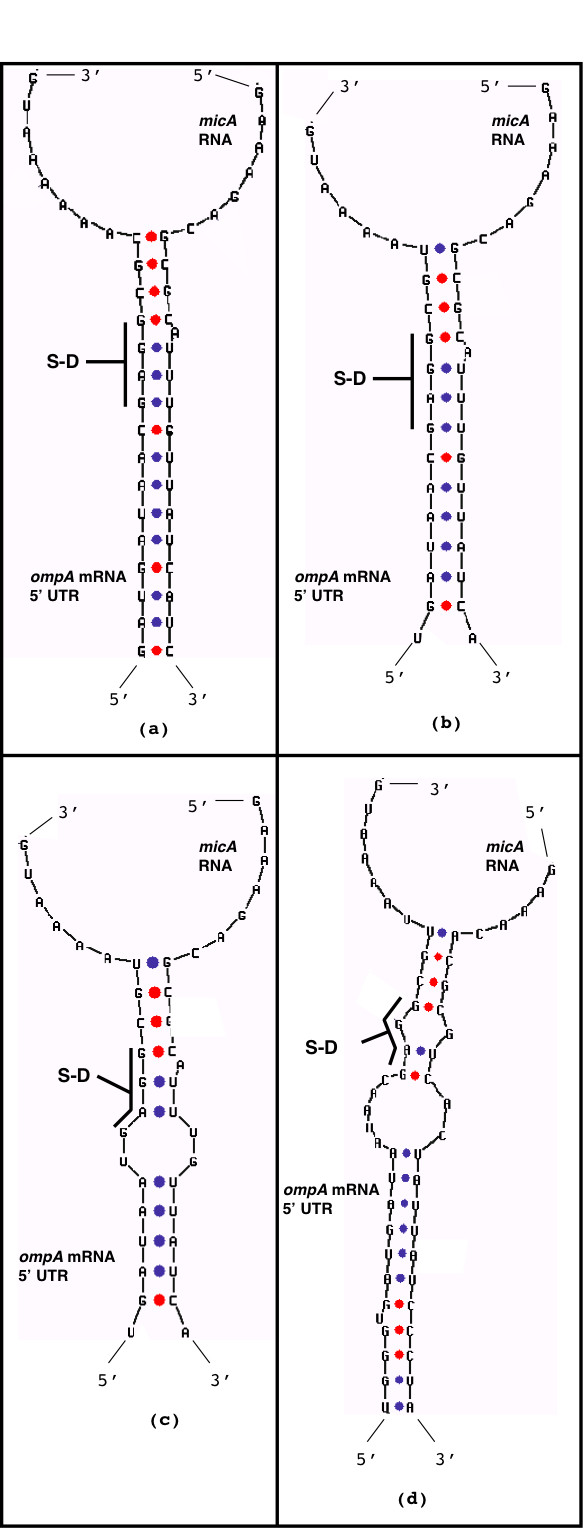
Secondary structure models of *micA *RNA/ompA mRNA 5' UTR interaction. a) *E. coli*, b) *Y. enterocolitica*, c) *Y. pestis*, d) *P. luminescens*. Note: the *ompA *mRNA 5' UTR and *micA *sequences of *Y. enterocolitica *were not previously published. In this study, *ompA *and *micA *gene sequences were identified in *Y. enterocolitica *by a blast search of the genome using the Sanger Institute blast server [41].

The *P. luminescens *sequence displays the major signatures of *micA *RNA, i.e., a rho-independent termination motif at the 3' end and the potential of 5' end sequences to base pair with region of the mRNA 5'UTR that covers the S-D sequence. Secondary structure models of the putative *P. luminescens micA *RNA together with *E. coli *and *Y. pestis micA *RNA structural models are in Figure S5, Supplement, Additional file 1. Figure 11 shows proposed *micA *RNA/*ompA *mRNA 5' UTR duplex interaction for *P. luminescens*, together with duplexes from related enterobacteria. All duplex models were obtained by the mfold program [26]. Although there are variations in duplex structures, the *P. luminescens *duplex shows close similarities to the other structures, e.g., shielding of the mRNA S-D ribosome binding site by the 5' end region of *micA *RNA, similarity in the length of the RNA/RNA duplex interaction and a similarity in 5' nucleotide position of *micA *RNA that participates in base pairing. Thus we propose that the sequence at positions 1451569 – 1451500 of the *P. luminescens *genome represents the *micA *gene homolog in *P. luminescens*.

By bioinformatics methods, Griffiths-Jones et al [42] and Hershberg et al [20] predicted and cataloged the small non-coding RNAs/RNA genes present in *E. coli *and related organisms. Seventeen are regulatory RNAs in *E. coli *(Table S2, Supplement, Additional file 1). Of the total small RNAs reported for *P. luminescens *on the Rfam website [43] (also D. Schones, Cold Spring Harbor Laboratory and N. Delihas, unpublished data), five genes encode regulatory RNAs (Tables S2 and S3, Supplement, Additional file 1). The work reported here adds *micA *to this list. Twenty-five *E. coli *ncRNAs are of undetermined function (Table S4, Supplement, Additional file 1) and *P. luminescens *shares four of these ncRNA genes.

## Discussion

*P. luminescens *evolved to proliferate in specialized environments and this poses interesting questions in terms of its gene composition. For free living bacteria, cell surface components and associated regulatory mechanisms are crucial to survival. The work here focuses on outer membrane porin protein genes and their regulatory RNA genes.

An *ompF*-type gene has been identified in *P. luminescens *as well as a putative *ompF *duplication (*ompF *paralog). The proposal for an *ompF *paralog is strongly supported by the mRNA 5' UTR compensatory base-pair changes, which maintain secondary structure signatures (Figure 6). The *ompF*-like gene was originally annotated as *ompN *in the *P. luminescens *genome, but there is no evidence for an *ompN *in *Photorhabdus*, either in chromosomal location or in the putative mRNA 5' UTR sequence. The homolog to *ompC*, which encodes the smaller porin has not been detected. No other porin protein genes, such as *phoE *[44] were found, but the specialized porin gene *lamB*, which encodes the protein involved in transport of maltose and maltodextrins across the cell envelope [45] has been annotated in *P. luminescens *[15]. In addition, *ompA*, which encodes a cell envelope protein that has multiple functions, was also previously annotated in *P. luminescens*.

OmpF is a generalized porin. It functions to allow for the intake of small nutrients into the cell and therefore serves an indispensable function. Therefore one would expect to find an *ompF*-type gene in *P. luminescens*. *ompF *is found in gamma subdivision enterobacteria whose genomes have been sequenced, including the obligate symbionts such as *Buchnera *and *Wiggleswothia *[46,47]. In *E. coli*, OmpF is one of the most abundant proteins in the cell. But perhaps each organism evolved its own porin properties such as conductance and pore size based on needs to thrive in specialized habitats and there may be nuances between *E. coli *and related bacterial OmpF functions.

OmpC is also a generalized porin, but may function primarily under conditions of high osmolarity [2]. OmpC may not be essential during either the symbiotic or pathogenic phases of *P. luminescens*. OmpA is a surface protein essential for the structural integrity of the cell envelope and as expected, is widely found in Gram-negative bacteria. The function of OmpN in *E.coli *is not known and it is uncertain whether *ompN *is an essential gene in other species. OmpN is expressed at very low levels in *E. coli strain BL21 *when grown in rich media [48], however levels in cells grown in minimal media were not determined. Interestingly, strain *E. coli O157:H7 EDL933 *appears to have a truncated *ompN *gene that codes for only123 amino acids out of the 377 amino acid sequence found in *E. coli K12*. *Salmonella enterica (subsp. enterica serovar Typhi str. CT18) *lacks an *ompN *gene at the *E. coli ompN *chromosomal site but has instead a transposase insertion sequence (unpublished). It appears that *ompN *is a pseudogene in *E. coli O157:H7 EDL933 *and it may not be uniformly present in species closely related to *E. coli*.

The proposed *ompF *duplication in *P. luminescens *(*plu1752*) does not appear to be a pseudogene. It codes for a nearly full length OmpF-type polypeptide, maintains a porin structural motif [49] and has upstream nucleotide sequences similar to three of the four OmpR binding sites and sequences almost identical to the -10 and -35 promoter region of *P. luminescens ompF*. Assuming *plu1752 *arose from *ompF*, it appears that the coding sequence has diverged from *ompF*, i.e., there is 59% amino acid sequence identity between *ompF *duplication and *ompF *coding regions.

Why is there a duplication of the *ompF*-type porin gene in *P. luminescens*? This may be related to requirements for small solute assimilation and a paralog of this porin may offer an advantage for survival of this organism in its specialized ecological niches. There are some differences between the *P. luminescens *putative *ompF *and *plu1752 *Loop 3 amino acid sequences but no apparent changes in net charge. Amino acid sequence differences in L-3 can affect pore properties [33,34]. The *plu1752 *putative mRNA 5' UTR and upstream OmpR binding region also have some changes. Therefore, it is possible *plu1752 *is regulated differently from the putative *ompF *and/or serves a more specialized function.

There is an apparent absence of a *micF *RNA gene homolog in *P. luminescens*. This suggests that post-transcriptional regulation of *ompF *expression is not essential for survival of this organism. In *E. coli*, *micF *expression is increased when the cell is under environmental stress conditions, e.g., exposure to toxic compounds, high temperature, cationic peptide antibiotics, or oxidative stress [3]. The confined life cycle of *P. luminescens*, involving only endosymbiotic and pathogenic phases, perhaps precludes exposure to these stress conditions. Thus *Photorhabdus *may have lost the *micF *gene when it evolved into an endosymbiont.

*OmpA *mRNA is degraded faster when *E. coli *cells enter stationary phase compared with log phase [9] and *micA *RNA appears to down-regulate *ompA *expression when cells enter stationary phase [10]. *P. luminescens *appears to have retained the *micA *gene. During the pathogenic phase, *P. luminescens *will go into stationary phase when insect nutrients are depleted [13]. Thus the proposed *micA *RNA may serve a similar function in *P. luminescens *as it does in *E. coli*. However, regulatory RNAs may not serve identical functional roles in organisms with different habitats. For example, *Serratia marcescens micF *responds to most but not all environmental factors that affect *micF *in *E. coli *[50].

*Photorhabdus *appears to have fewer *E. coli *regulatory ncRNA genes than its close relatives (Table S2, Supplement, Additional file 1). Important is the absence of *rygA *and *rygB*, two ncRNA genes that are proposed to regulate multiple outer membrane proteins in response to environmental conditions [51]. However, further searches are needed to definitively show absence of these genes in *P. luminescens*. On the other hand, *P. luminescens*, because of its complex life cycle, may have its own group of regulatory ncRNAs. For example, a small RNA has been hypothesized to regulate pathways leading to either symbiosis or pathogenicity [52]. In the extreme case of the obligate endosymbionts such as *Buchnera *and *Wiggleswothia*, only the *E. coli *housekeeping RNA genes are reported and there were no regulatory RNA genes found [42] (and D. Schones and N. Delihas, unpublished). Control of gene expression by RNA may be more prevalent in cells where survival is dependent on the ability to make rapid adjustments in response to environmental stress. Bacterial regulatory ncRNA genes probably evolved to help the organism survive under different environmental conditions and stress-related factors and these genes may not be present in organisms that live in more protected environments. Parallel to this, loss of transcriptional regulators has been reported for the obligate symbiont *Buchnera*, which grows only in the aphid host [53].

## Conclusions and perspectives

Analysis of the *P. luminescens *genome described here and by others shows essential outer membrane protein genes *ompF*, *ompA *and a proposed duplication of *ompF*. The post-transcriptional regulatory RNA gene *micA *appears to also be present, but the *ompF *regulatory counterpart *micF *is not evident. The presence or absence of certain ncRNA genes in *Photorhabdus *appears consistent with the rationale for a need or lack of need. On the other hand, new RNA genes may have evolved to help the organism thrive in its environment. Further analyses of the *P. luminescens *genome by bioinformatics and/or microarray analyses may reveal regulatory ncRNA genes or gene transcripts that may be unique to *Photorhabdus *and closely related species that function in the pathogenic phase. Along with the marked increase in virulence genes [13-15], genes that encode both regulatory proteins and regulatory RNAs may also have evolved and/or were acquired to increase the pathogenicity of the organism.

Two pertinent projects are underway. The genomes of the related symbiont and/or insect pathogens, *Xenorhabdus nematophilia *[54] and *Photorhabdus asymbiotica *[55] are currently being sequenced. When completed, these sequences may add to and/or complement the assessment of outer membrane porin genes and RNA regulatory genes presented here.  *P. asymbiotica *is of particular interest since in some instances it is found to cause human opportunistic infections. This organism offers exciting opportunities to further assess virulence factors and their regulation.

## Methods and materials

To search for outer membrane protein and regulatory RNA genes, the BLAST GenBank site of the National Center for Biotechnology Information [56] was used. Genome searches were with the BLAST with microbial genomes page [57]), with the exception of the *Yersinia enterocolitica *genome where the Welcome Trust Sanger institute site [58] was employed. The Entrez cross-database search page [16] was used to find nearest neighbor genes.

RNA secondary structure modeling was performed with the Zuker and Turner Mfold, version 3.2 [59,26]. Standard constraint parameters were used with the following: maximum interior/bulge loop size was 30, maximum asymmetry of an interior/bulge loop was 30 and there was no limit on maximum distance between paired bases. mRNA/ncRNA duplex structures were obtained by positioning the two individual RNAs 5' to 3' one after the other starting with the mRNA first and adding ten Ns between the two polynucletides. RNA secondary structures were displayed with the New Structure Viewing Options and figures in this paper were modified from graphic dispay by Output of sir_graph by D. Stewart and M. Zuker.

Nucleotide sequences were aligned with the DNASTAR MegAlign alignment program [60]. Parameters used were that of J. Hein with gap penalty 11, gap length, 3; ClustalV, with gap penalty 10, gap length 10 or ClustalW with gap penalty 15, gap length, 6.66. Percent identities and consensus sequences were based on DNASTAR program.

Proposed 5' start of RNAs was based on alignment with known 5' start sites of homologous RNAs.

### Search for *micF *RNA primary and secondary structure motifs in *P. luminescens*

The strategy used to search for a putative *micF *RNA in *P. luminescens *was to scan the genome using the conserved 13 nt 5' end *micF *sequence, i.e., 5'G_1_CTATCATCATTA_13_3' as well as variations of this sequence. Variations included T at position 2, T at positions 6 and 9, and in addition, a total of 4 random substitutions. A different first pattern that provides perfect complementarily to the *ompF *mRNA 5' UTR was also employed: 5'G_1_TTTCATCATTATT_14_3'. Variations included a total of four random substitutions and also allowing for the insertion of an A residue randomly between the 3rd and 10th base of the pattern. Additional constraints consisted of a rho- independent termination pattern situated 35–85 bp downstream the two basic 5' end patterns shown above. The parameters used for the terminal rho- independent structure were a stem-loop followed by at least four T residues. The stem was 4–15 bp with a minimum of three G-C pairs, the loop 3–8 bases, and the maximum folding energy of loop was -9 Kcal/mol. Scans for the termination motif were performed after the initial identification of the two patterns shown above.

For scanning the *P. luminescens *genome, the perl programing language was used. Additional scans were performed for -10 and -35 promoter sequences as described in Supplement, Additional file 1. To avoid 0 values, discounting for the probabilities in the consensus sequences were applied. Jeffrey Perk's law was used [61]:

Jeffey Perk's law: P(w) = (C(w)+1/2)/(N+B/2),

where P is the assigned probability, w is a DNA character assignment, C(w) is the frequency of the character in the consensus table for the specific position, N is the number of training sequences used for the creation of the consensus table and B is the number of possible values for our character i.e., 4.

## Abbreviations

### Nucleic acids

nt, nucleotide; N, any of the four ribo- or deoxyribo-nucleotides A,C,G,U, or T; Mb, 10^6 ^base pairs (megabases); S-D, Shine-Dalgarno ribosome binding site; ncRNA, non-coding RNA.

### Organisms

B.a. *Buchnera aphidicola*; E.c., *Escherichia coli*; Er.c. *Erwinia carotovora*; P.l. *Photorhabdus luminescens*; S.t., *Salmonella typhimurium *or *Salmonella typhi*; S.m., *Serratia marcescens*; Shs, *Shigella sonnei*; W.g., *Wigglesworthia glossinidia*; Xn, *X. nematophilia*; Y.e.,*Yersinia enterocolitica*; Y.p., *Yersinia pestis*; Yptb, *Y. pseudotuberculosis*;

### Proteins

OmpA, outer membrane protein A; OmpC, outer membrane protein C; ompN, outer membrane protein N; OmpF, outer membrane protein F.

## Competing interests

The author(s) declare that they have no competing interests.

## Authors' contributions

D.P. designed bioinformatics search programs and obtained data for *micF*. ND designed overall experimental approaches and obtained data for outer membrane proteins and regulatory RNAs.

## Reviewers comments'

### Reviewer's report 1

Tal Dagan (nominated by Dan Grauer), Institut fuer Botanik III Heinrich-Heine Universitaet Duesseldorf Universitaetsstr. 1, 40225 Duesseldorf, Germany

Second review following revisions: The manuscript by Papamichail & Delihas deals with the prediction of outer membrane proteins (OMP) and their regulators in the genome of *Photorhabdus luminescens*. The genome of *Escherichia coli*, a close relative of *P. luminescens*, contains three major outer membrane proteins that are regulated by trans-coded antisense RNAs. Using bioinformatics tools, Papamichail & Delihas search for the existence of similar genes in the genome of *P. luminescens*.

Following the first review iteration, bioinformatics analyses have been added to improve the manuscript. In its current state, the study presents a convincing evidence for the existence/loss of OMP in *P. luminescens*.

Remarks:

- In figure 1, there are two unmarked objects: a blue one in *E. coli*, and pink one in *S. typhimurium*. Following the reply to the first review I now know which are they, but I think it is important to repot the information also to the reader (i.e., in the figure's legend).

- The presented alignments would be clearer in PrettyAlign format, or any other tool/format that presents an alignment with color-coded animo-acids. Such format enables the reader an immediate overall perception of the sequences' conservation degree.

- Citing links that oblige the user to enter some detail (such as CDD and other NCBI links) may be problematic, mainly because the cited databases may be updated after the publication of the manuscript. I would recommend saving the query result and adding it as supplementary material.

### Reviewer's report 2

Mikhail S. Gelfand (with additional advice from Anna Gerasimova), Institute for Information Transmission Problems, RAS11 Bolshoy Karetnyper. 19, Moscow, GSP-4, 127994, Russia

Second review following revisions: We have no further comments. The paper is much improved following revision.

### Author's response

Drs. Gelfand's and Gerasimova's initial comments were that the manuscript was interesting but limited in that a global assessment of ncRNAs as well as phylogenetic relations of *ompFs *were needed. These have been included in the Supplement.

### Reviewer's report 3

J Peter Gogarten, University of Connecticut, Biology/Physics Building, Rooms 404/426/427 Unit 3125 91 North Eagleville Road Storrs CT 06269-3125 USA

Second review following revisions: The authors describe a search of the *Photorhabdus luminescens *genome for outer membrane proteins and for their trans encoded small RNA regulators. In addition to the previously recognized *ompA *homolog, the authors identify two *ompF *homologs in the *P. luminescens *genome that evolved from a gene duplication. A search for small regulatory RNAs only identifies a *micA *homolog. The authors discuss their findings in light of the *P. luminescens *lifecycle.

Minor criticisms and suggestions were made [in the first review] and the have authors satisfactorily addressed these.

## Websites references

National Center for Biotechnology Information (NCBI) GenBank sites:









BioCyc Database Collection





*mfold server *: 1995-2006, Michael Zuker, Rensselaer Polytechnic Institute:



Donald Danforth Plant Science Center:



The Welcome Trust Sanger Institute







DNASTAR, Inc



## Accession numbers of bacterial strains

NC_004061*Buchnera aphidicola str. Sg*

NC_00443*Escherichia coli CFT073*

NC_000913*Escherichia coli K12*

NC_002655*Escherichia coli O157:H7 EDL933*

NC_005126*Photorhabdus luminescens subsp. laumondii TTO1*

NC_003197*Salmonella typhimurium LT2*,

NC_003198*Salmonella enterica (subsp. enterica serovar Typhi str. CT18)*

NC_004337*Shigella flexneri *2a str. 301,

NC 004741*Shigella flexneri 2a str. 2457T*,

NC_003143*Yersinia pestis CO92*

NC_006155*Yersinia pseudotuberculosis IP 32953*

## Supplementary Material

Additional File 1 a. OmpF Loop 3 b. Search for a putative *P. luminescens micF *RNA c. Promoter search methods d. Figures: *ompA *mRNA 5' UTR and *micA *RNA e. Non-coding RNAs in *E. coli*-related bacteria Click here for file
