# Supplementary material for: Outer membrane protein genes and their small non-coding RNA regulator genes in Photorhabdus luminescens
Source: Biol Direct. 2006 May 22;1:12. doi: 10.1186/1745-6150-1-12 (PMC1489923; doi:10.1186/1745-6150-1-12)
Supplement: Additional File 1 — a. OmpF Loop 3 b. Search for a putative P. luminescens micF RNA c. Promoter search methods d. Figures: ompA mRNA 5' UTR and micA RNA e. Non-coding RNAs in E. coli-related bacteria [file 1745-6150-1-12-S1.pdf]

“Outer membrane protein genes and their small non-coding RNA regulator genes in *Photorhabdus luminescens*”

Dimitris Papamichail and Nicholas Delihas

SUPPLEMENT

a. OmpF Loop 3

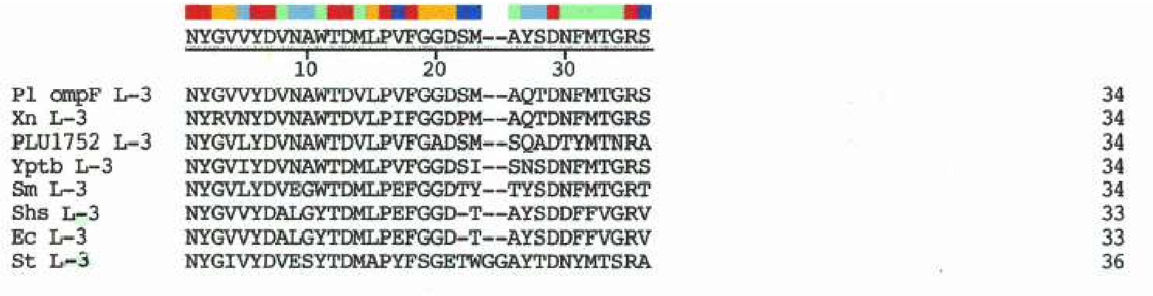

Figure S1. Alignment of Loop 3 (L-3) amino acid sequences of porins using the DNASTAR ClustalW program. Positions 123-155 of *E. coli* OmpF used as guide. Pl, *P. luminescens*, Xn, *X. nematophilia*; PlU1752, *P. luminescens*: Yptb, *Y. pseudotuberculosis*; Sm, *S. marcescens*; Shs, *S. sonnei*; ec, *E. coli*; St, *S. typhi*. All porin L-3 refer to OmpF except OmpF-like (Pl and Xn) and OmpN (Ec).

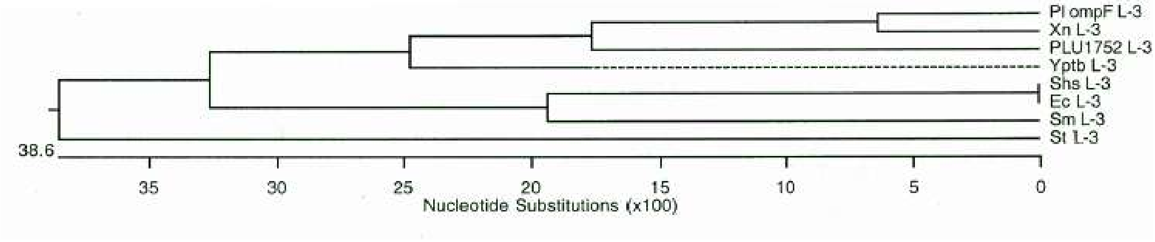

Figure S2. Phylogenetic tree of Loop 3 (L-3) amino acid sequences determined by DNASTAR program. Abbreviations same as in Figure S1.

Table S1

Percent identity L-3

	Xn L-3	PLU1752 L-3	Yptb L-3	Sm L-3	Shs L-3	Ec L-3	Ec OmpN L-3	St L-3
Pl ompF L-3	**88**	**77**	**82**	**68**	**59**	**59**	**68**	**50**
Xn L-3	*	**68**	**74**	**65**	**53**	**53**	**62**	**44**
PLU1752 L-3	*	*	**71**	**59**	**44**	**44**	**62**	**50**
Yptb L-3	*	*	*	**74**	**59**	**59**	**74**	**50**
Sm L-3	*	*	*	*	**68**	**71**	**82**	**56**
Shs L-3	*	*	*	*	*	**97**	**64**	**46**
Ec L-3	*	*	*	*	*	*	**64**	**49**
Ec OmpN L-3	*	*	*	*	*	*	*	**56**

Abreviations: Pl, *P. luminescens*, Xn, *X. nematophilia*; PLU1752, *P. luminescens*: Yptb, *Y. pseudotuberculosis*; Sm, *S. marcescens*; Shs, *S. sonnei*; Ec, *E. coli*; St, *S. typhi*. All porin L-3 refer to OmpF except OmpF-like (Pl and Xn), Pl proposed paralog (PLU1752), and OmpN (Ec).

Table S1. Percent identity of Loop 3 (L-3) porin amino acid sequences determined by DNASTAR program. Abbreviations same as in Figure S1.

b. Search for a putative *P. luminescens micF* RNA

In the search for a putative *P. luminescens micF* RNA, the "fifth positive" sequence (described in the main text under the section **a. *ompC* and *micF***) is found in an intergenic region. It is 414 bp from the 5' side of the *ptsH* gene and 35 bp from the 3' side of the *cysK* gene and is located at positions 1674515-1674620 of the *P. luminescens* genome.

A promoter search was also performed for the *P.luminescens* "fifth positive" sequence. The results are described in terms of a p-value (the probability that the examined sequence appears by chance). The most prominent promoter candidate for the "fifth positive sequence" had a p-value of 2.0×10^{-3} and was calculated for the P-35 at -37 and a spacer of 17 bp between the –35 and –10 sequences. For the *E.coli micF* promoter (reference S1) the calculated p-value is 3.3×10^{-3} (P-35 at –36 and spacer of 17 bp). Although the "fifth positive" sequence provides a promoter probability in the range of that for *E. coli micF*, the *micF* promoter produces a weak signal. The statistical methods used are described below.

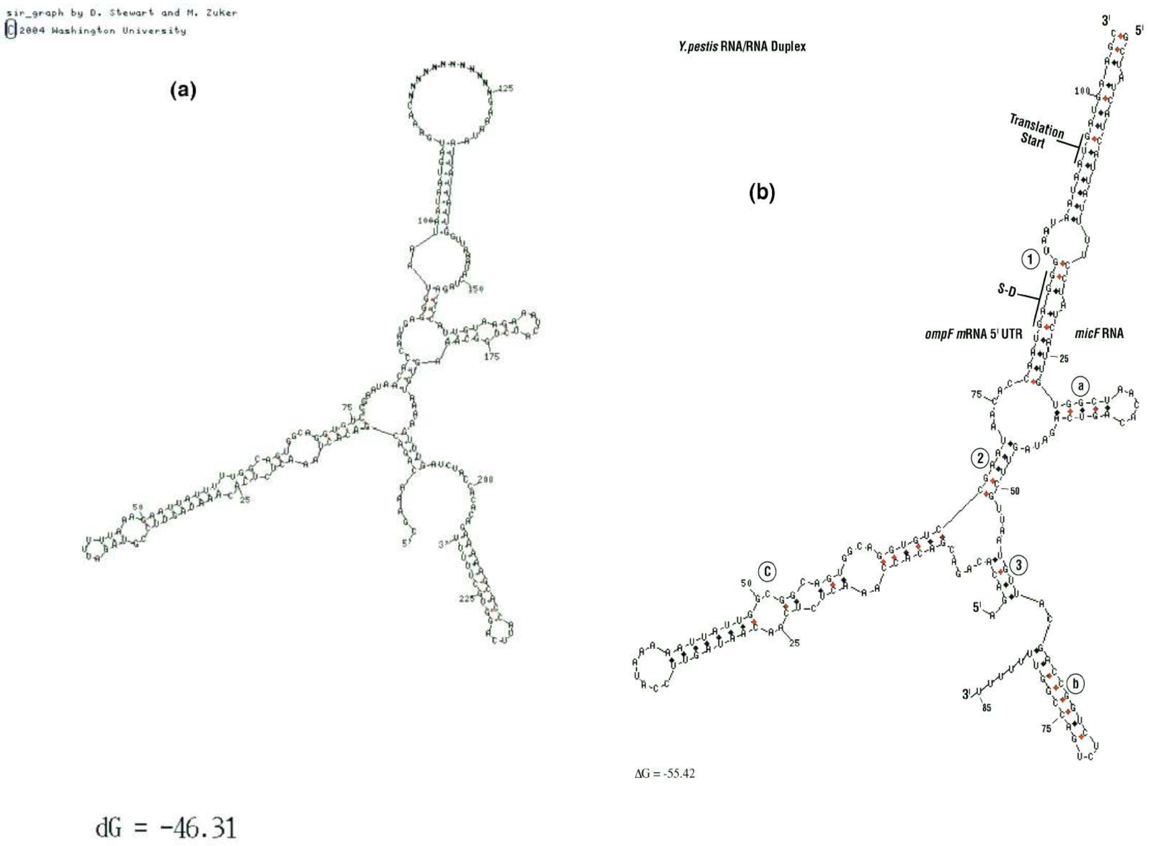

Figure S3. a) RNA/RNA secondary structure model of the “fifth positive” sequences, b) *Y. pestis micF* RNA/*ompF* mRNA 5’ UTR duplex model, reproduced from reference S2.

Supplement Figure S3a shows a model of the RNA/RNA duplex structure of the “fifth positive” sequence with *P. luminescens ompF* mRNA 5’ UTR. Figure S3b (reproduced from reference S2) shows the *Y. pestis micF* RNA/*ompF* mRNA 5’ UTR duplex as a comparison. This structure has the characteristics of *micF/ompF* RNA/RNA duplexes with the exception of “blunt ends” which is characreristic of duplexes from all bacteria known to have a *micF* gene. Thus although we have discounted this sequence as a potential *micF* in *P. luminescens*, we cannot rule out that the “fifth positive sequence” encodes a small RNA as it has possible promoter and termination signals.

c. Promoter search methods

In order to locate possible P-35 and P-10 promoter sites for candidate sequences, a search was performed in the region upstream of the sequence, starting at -50 and ending at -30 for the P-35 promoter. The spacer between the P-35 and P-10 promoter allowed for a length of 15-19 bp.

To calculate p-values (probability that the examined sequence appears by chance) and to examine the significance of the promoter findings, the base

frequency distributions in the consensus hexamers as compiled in reference (S3) was used. Initially, the probability of the two hexamers appearing in a specific location (under the constraints previously specified) is calculated as the geometric mean of the product of the individual frequencies of all bases of the candidate promoters at this location. In order to calculate a p-value for this probability, a pre-compiled sorted table of all possible 4^{12} values of probabilities for each hexamer pair that could comprise our two promoters is consulted, in order to locate the value of the probability for the location we are examining. The p-value is then calculated as the number of values equal or higher than the one we calculated, over the total number of values (4^{12}).

Although the p-values are significant, they become less significant if corrected for the number of samples, e.g. Bonferroni correction (reference S4), since a variety of possible positions for the P-35 promoter and five values, 15-19 for the spacer between the two promoters were considered. This is true for the known promoter site for the *E. coil micF*. Thus this accounts for attributing the *E.coli micF* as a weak signal.

d. Figures: *ompA* mRNA 5' UTR and *micA* RNA

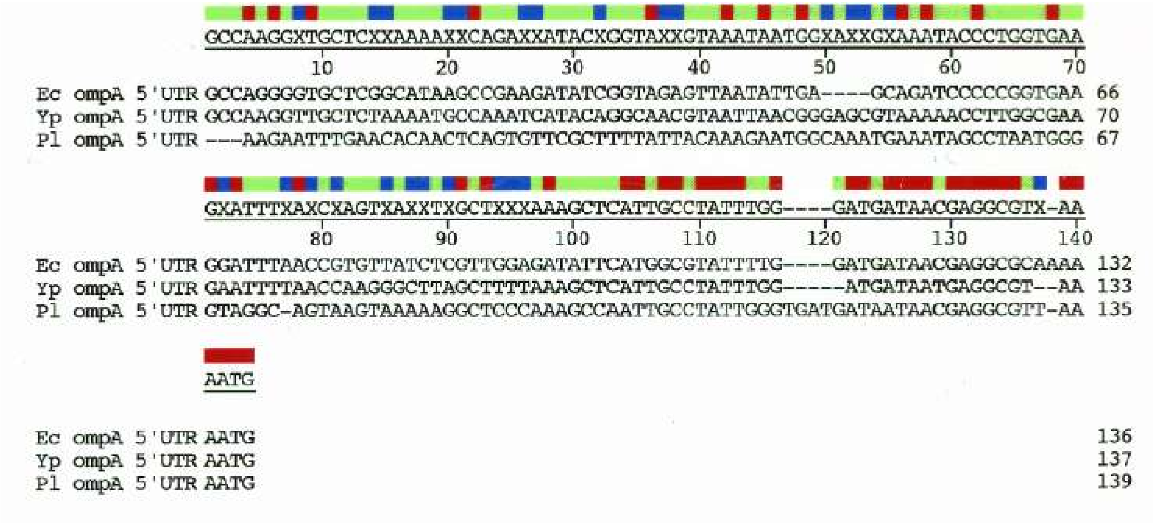

Figure S4. Alignment of *E. coli, Y. pestis, P. luminescens ompA* 5' UTR sequences. The ATG at positions 142-144 is the coding start site.

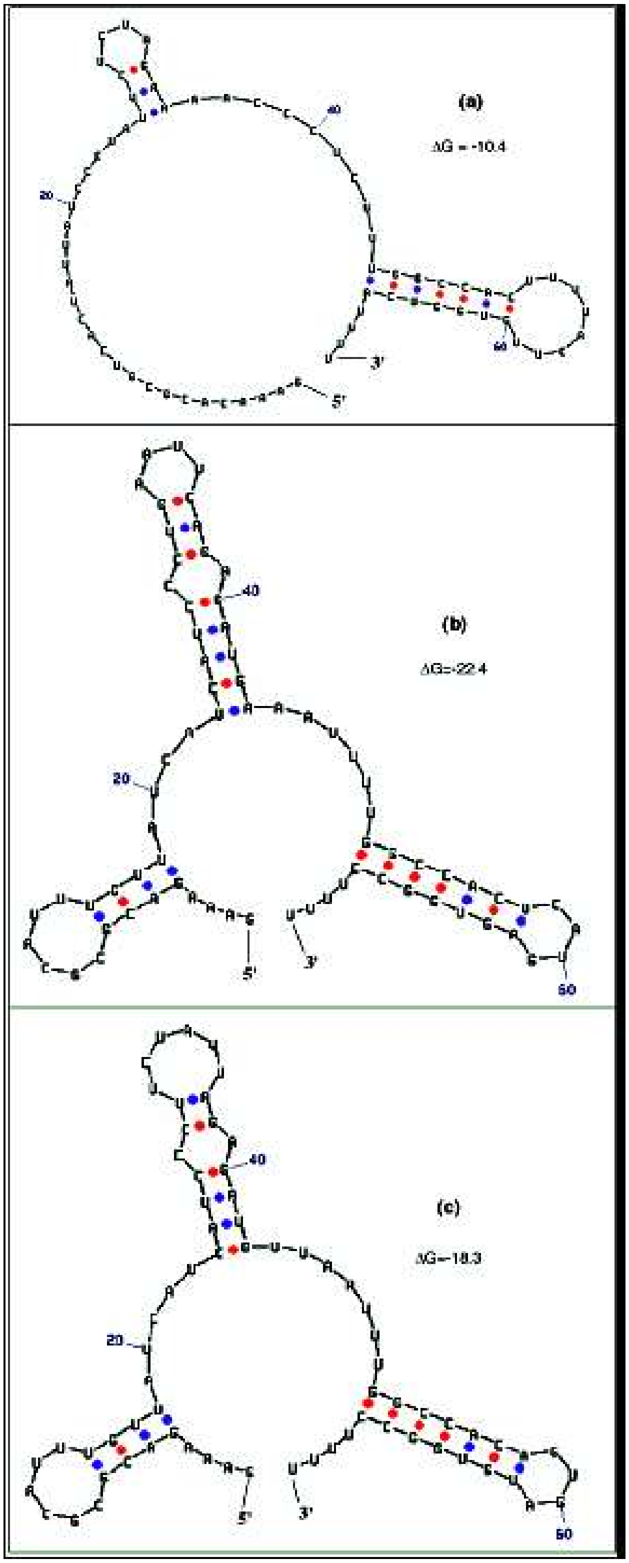

Figure S5. Secondary structure models of *micA* RNA: a) *P. luminescens*; b) *Y. pestis*; c) *E. coli*.

e. Non-coding RNAs in *E. coli*-related bacteria

A global picture of *E. coli* regulatory ncRNA/RNA genes and the reported presence or absence from related gamma proteobacteria is in Table S2. Table S4 shows ncRNAs whose functions have not yet been determined. The data shown are primarily from Rfam as well as unpublished data by D. Schones and N. Delihas. Six *E. coli* regulatory ncRNA genes appear to be in *P. luminescens* and their functions are described in Table S3. *P. luminescens* shares four of the twenty five *E. coli* ncRNA genes of undetermined function (Table S4).

Table S2

Regulatory non-coding RNAs*

**ncRNA/RNA gene**	**E.c.**	**S.t.**	**Y.p.**	**Er.c.**	**P.l.**	**W.s.**	**B.a.**
RygA[1]	RygA	RygA	RygA	RygA	-	-	-
RygB[1]	RygB	RygB	RygB	RygB	-	-	-
RydC	RydC	RydC	-	-	-	-	-
GadY	GadY	-	-	-	-	-	-
MicC	MicC	MicC	-	-	-	-	-
CsrC	CsrC	CsrC	CsrC	-	-	-	-
RyhB	RyhB	RyhB	RyhB	RyhB	RyhB	-	-
DicF	DicF	DicF	-	-	-	-	-
OxyS	OxyS	OxyS	-	-	-	-	-
MicF	MicF	MicF	MicF	?	-	-	-
GcvB	GcvB	GcvB	GcvB	GcvB	GcvB	-	-
Spot42	Spot42	Spot42	Spot42	Spot42	Spot42	-	-
CsrB	CsrB	CsrB	CsrB	CsrB	CsrB	-	-
DsrA	DsrA	DsrA	-	-	-	-	-
RprA	RprA	RprA	RprA	-	-	-	-
6S	6S	6S	6S	6S	6S	-	-
micA	micA	micA	micA	micA	micA	n.d.	n.d.
**Total**	**17**	**16**	**11**	**8**	**6**	**0**	**0**

Table S2. *E. coli* regulatory nc RNAs in gamma proteobacteria. All data from Rfam website (S6) except *micA*. *micA* in E.c.,S.t.,TY.p.,Er.c. from Udekwu et al (S7). *micA* in P.l., this determination. Abbreviations: E.c. *Escherichia coli K-12 MG1655*; S.t., *Salmonella enterica subsp. enterica serovar Typhi str. CT18*; Y.p., *Yersinia pestis strain CO92*; Er.c. *Erwinia carotovora subsp. atroseptica SCRI104*; P.l. *Photorhabdus luminescens subsp. laumondii* TTO;1 W.g., *Wigglesworthia glossinidia endosymbiont of Glossina brevipalpis*; B.a. *Buchnera aphidicola str. APS (Acyrthosiphon pisum.)*

Table S3

Regulatory ncRNAs in *P. luminescens*

RNA gene	Function[1]
gcvB	regulates dipeptide and oligopeptide transport
spf (Spot42)	regulates the galactose operon
ryhB	down-regulates genes that encode iron containing proteins
6S RNA	regulates sigma factors 70 and S during stationary phase
csrB	regulates glycogen biosynthesis
micA	down-regulates the level of *ompA* mRNA at stationary phase

[1]As described by Griffiths-Jones et al (S6).

Table S3. Regulatory ncRNAs and functions in *P. luminescens*.

Table S4

Non-coding RNAs – function unknown*

ncRNA/RNA gene	E.c.	S.t.	Y.p.	Er.c.	P.l.	W.s.	B.a.
sroH	sroH	-	-	-	-	-	-
sroE	sroE	sroE	-	-	-	-	-
sroD	sroD	sroD	-	-	-	-	-
sroC	sroC	sroC	-	-	-	-	-
sroB	sroB	sroB	sroB	-	-	-	-
tke1	tke1	tke1	tke1	-	tke1	-	-
t44	t44	-	t44	t44	t44	-	-
ryfA	ryfA	ryfA	-	-	-	-	-
IS128	IS128	-	-	-	-	-	-
IS102	IS102	-	-	-	-	-	-
C0343	C0343	C0343	-	-	-	-	-
C0299	C0299	-	-	-	-	-	-
rydB	rydB	rydB	-	-	-	-	-
C0719	C0719	-	-	-	-	-	-
C0465	C0465	-	-	-	-	-	-
IS061	IS061	-	-	-	-	-	-
RyeE	RyeE	RyeE	RyeE	-	-	-	-
RyeB	RyeB	RyeB	RyeB	RyeB	-	-	-
RybB	RybB	RybB	RybB	RybB	RybB	-	-
SraC_RyeA	SraC_RyeA	SraC_RyeA	SraC_RyeA	SraC_RyeA	-	-	-
SraJ	SraJ	SraJ	-	-	-	-	-
SraG	SraG	SraG	SraG	SraG	SraG	-	-
SraH	SraH	SraH	SraH	-	-	-	-
SraD	SraD	SraD	SraD	SraD	-	-	-
SraB	SraB	SraB	-	-	-	-	-
**Total**	**25**	**17**	**10**	**6**	**4**	**0**	**0**

All data according to Rfam website (S6)

Abbreviations: E.c. *Escherichia coli K-12 MG1655*; S.t., *Salmonella enterica subsp. enterica serovar Typhi str. CT18*; Y.p., *Yersinia pestis strain CO92*; Er.c. *Erwinia carotovora subsp. atroseptica SCRI104*; P.l. *Photorhabdus luminescens subsp. laumondii* TTO;1 W.g., *Wigglesworthia glossinidia endosymbiont of Glossina brevipalpis*; B.a. *Buchnera aphidicola str. APS (Acyrthosiphon pisum.)*

Table S4. *E. coli* ncRNAs of undetermined function and presence in gamma proteobacteria. Abbreviations as in Table S2.

Supplement References:

S1. Coyer J, Andersen J, Forst SA, Inouye M, Delihas N: ***micF* RNA in ompB mutants of *Escherichia coli*: different pathways regulate micF RNA levels in response to osmolarity and temperature change**. *J Bacteriol* 1990, **172**:4143-4150.

S2. Delihas N: **Annotation and evolutionary relationships of a small regulatory RNA gene micF and its target ompF in Yersinia species**. *BMC Microbiol* 2003, **3**:13.

S3. Lisser S Margalit H: **Compilation of *E.coli* mRNA promoter sequences**, *Nucleic Acids Research* 1993, **21**:1507-1516.

S4. Bonferroni, C. E. **Il calcolo delle assicurazioni su gruppi di teste,** *Studi in Onore del Professore Salvatore Ortu Carboni.* Rome: Italy, pp. 13-60, 1935

S5. Guillier M, Gottesman S: **Remodelling of the *Escherichia coli* outer membrane by two small regulatory RNAs**. *Mol Microbiol* 2006, **59**:231-247.

S6. Griffiths-Jones S Moxon S Marshall M Khanna A Eddy SR Bateman A: **Rfam: annotating non-coding RNAs in complete genomes.** *Nucleic Acids Res* 2005, **33**(Database issue):D121-124.

S7. Udekwu KI, Darfeuille F, Vogel J, Reimegard J, Holmqvist E, Wagner EG: **Hfq-dependent regulation of OmpA synthesis is mediated by an antisense RNA**. *Genes Dev*. 2005, **19**:2355-2366.
